# Molecular Advancements Establishing Chlamydomonas as a Host for Biotechnological Exploitation

**DOI:** 10.3389/fpls.2022.911483

**Published:** 2022-06-29

**Authors:** Michael Schroda, Claire Remacle

**Affiliations:** ^1^Molecular Biotechnology and Systems Biology, TU Kaiserslautern, Kaiserslautern, Germany; ^2^Genetics and Physiology of Microalgae, InBios/Phytosystems Research Unit, University of Liege, Liege, Belgium

**Keywords:** Chlamydomonas, MoClo, genome editing, CRISPR/Cas9, nuclear transgene expression

## Abstract

*Chlamydomonas reinhardtii* is emerging as a production platform for biotechnological purposes thanks to recent achievements, which we briefly summarize in this review. Firstly, robust nuclear transgene expression is now possible because several impressive improvements have been made in recent years. Strains allowing efficient and stable nuclear transgene expression are available and were recently made more amenable to rational biotechnological approaches by enabling genetic crosses and identifying their causative mutation. The MoClo synthetic biology strategy, based on Golden Gate cloning, was developed for Chlamydomonas and includes a growing toolkit of more than 100 genetic parts that can be robustly and rapidly assembled in a predefined order. This allows for rapid iterative cycles of transgene design, building, testing, and learning. Another major advancement came from various findings improving transgene design and expression such as the systematic addition of introns into codon-optimized coding sequences. Lastly, the CRISPR/Cas9 technology for genome editing has undergone several improvements since its first successful report in 2016, which opens the possibility of optimizing biosynthetic pathways by switching off competing ones. We provide a few examples demonstrating that all these recent developments firmly establish Chlamydomonas as a chassis for synthetic biology and allow the rewiring of its metabolism to new capabilities.

## Introduction

This mini-review provides a brief overview of recent developments that are turning the unicellular green alga *Chlamydomonas reinhardtii* into a flagship for algal biotechnology. These developments include strain improvement, findings that improve nuclear transgene expression, the Modular Cloning (MoClo) approach, and CRISPR/Cas9-mediated genome editing. For detailed compilations of established genetic tools and strains, we refer readers to recent comprehensive reviews (e.g., Jinkerson and Jonikas, 2015; Scaife and Smith, 2016; Kong et al., 2019; Salomé and Merchant, 2019; Schroda, 2019; Zhang et al., 2021).

Nuclear transformation is a prerequisite for strain improvement by genetic engineering. Chlamydomonas was one of the first microalgae for which an efficient transformation of the nuclear genome was established (Kindle, 1990). Transformation was based on a simple method where the foreign DNA was inserted in the nuclear genome after vortexing the recipient cells together with glass beads and DNA. Later on, efficient transformation was achieved using electroporation (Shimogawara et al., 1998). Two markers were used: the *ARG7* marker encoding argininosuccinate lyase, allowing the restoration of autotrophy of an *arg7* mutant cell line and the *NIT1* marker, encoding nitrate reductase, allowing the assimilation of nitrate in a *nit1* mutant strain (Debuchy et al., 1989; Kindle et al., 1989). Other markers have been developed thereafter, the most popular ones being the *aph7″* and *aphVIII* markers, conferring resistance to hygromycin B and paromomycin, respectively (Sizova et al., 2001; Berthold et al., 2002). Despite early successful nuclear transformation and publication of the nuclear genome sequence (Merchant et al., 2007), Chlamydomonas has failed to emerge as a chassis for biotechnological applications. The main reason is that Chlamydomonas efficiently silences transgenes. The mechanisms underlying efficient transgene silencing were dissected and it turned out that it is largely mediated at the nucleosome level involving histone modifications and associated chromatin compaction as well as unfavorable nucleosome positioning at the level of the newly added transgenes (Schroda, 2019). Recently, several strategies were developed to circumvent this problem and thereby establish Chlamydomonas as a chassis for synthetic biology with the goal of switching its metabolism to new capabilities.

## Strains With Efficient and Stable Nuclear Transgene Expression

In 2009, a genetic screen was developed allowing the selection of two strains, named UVM4 and UVM11, which express transgenes at high levels (Neupert et al., 2009). These strains were successfully used for high-level expression of various transgenes (e.g., Lauersen et al., 2015; Crozet et al., 2018; Durante et al., 2019). However, they can hardly be crossed, implying that each transgene must be inserted by transformation *via* different selection markers. This diminishes the number of genetic traits that can potentially be combined and increases the chance that one of the transformation events causes an undesired genetic defect due to the random insertion of the cassette. Furthermore, since the UVM strains lack a cell wall, they are less robust, which is not desirable for biotechnological purposes. Indeed, scaling up the production of algal compounds requires the cultivation of cells in large quantities, either in photobioreactors or open ponds, both of which impose shearing forces that can be detrimental to wall-less strains. The causative mutations in UVM4 and UVM11 were shown to affect the same locus encoding a Sir2-type histone deacetylase (SRTA; Neupert et al., 2020). In addition, a UVM11 strain with a cell wall and restored mating-capacity was generated, which opens the possibility of transferring transgenes by crosses and large-scale cultivation (Neupert et al., 2020). To alleviate the mentioned disadvantages of the UVM4 and UVM11 strains, another walled strain with high transgene expression and mating capacity was recently isolated and awaits to be used by the Chlamydomonas community (Dementyeva et al., 2021).

## Transgene Design: Promoters, 5’ UTRs and 3’ UTRs

The hybrid *HSP70A/RBCS2* (*AR* promoter) (Schroda et al., 2000) and the *PSAD* promoter (Fischer and Rochaix, 2001) have consistently shown robust expression of nuclear transgenes and are therefore widely used. They are considered as benchmarks for testing other promoters including synthetic ones (Scranton et al., 2016; Einhaus et al., 2021) but comparisons are always difficult since the sequences of the constructions used in the different reports vary and promoter performances vary with the transgene used. In addition, inducible promoters are available that can be desirable to control the production of compounds with toxic effects (Salomé and Merchant, 2019; Schroda, 2019). An alternative is the use of riboswitches. In Chlamydomonas and other eukaryotes, they are present in two genes involved in thiamine biosynthesis (*THI4* and *THIC*) and respond to thiamine pyrophosphate (TPP). The riboswitch of the *THI4* gene is a small RNA sequence (~170 bp, aptamer) located in the first intron of the 5’ UTR. When no thiamine is supplied, the intron is spliced and translation of the transcript leads to the active THI4 enzyme. When thiamine is supplied in the medium, the level of TPP rises and binds to the intron in the *THI4* mRNA, which leads to alternative splicing, demasking an alternative ATG initiation codon and an in frame STOP codon. A small protein is then produced that interferes with translation of the native THI4 (Mehrshahi et al., 2020). A synthetic riboswitch cassette comprising the intronic region with the aptamer was designed and shown to regulate the expression of a transgene (casbene synthase) involved in the biosynthesis of terpenes (Mehrshahi et al., 2020).

The untranslated regions (UTR) at the 5′ and 3′ ends of the transcripts should also be considered when designing transgenes. Einhaus et al., (2021) noticed an unexpected synergy between the 5’ UTR of *PSAD* and its chloroplast transit peptide sequence, which suggests that this sequence could provide accessibility to cis-regulatory elements and the positioning of the nucleosomes around the transgene (Einhaus et al., 2021). The first 3’ UTR used was that of the *RBCS2* gene (Stevens et al., 1996) but others, like the *FDX1* or *RPL23* terminators, better supported high-level transgene expression (López-Paz et al., 2017; Einhaus et al., 2021). A systematic comparison of nine terminators was recently published which expands the panel of 3’ UTRs that can be used (Geisler et al., 2021). Testing these terminators with different promoters, in different host strains and for different transgenes gave similar transformation efficiencies (Geisler et al., 2021), showing that the terminators tested are not by themselves greatly influencing the rate of transformation.

## Transgene Design: The Coding Sequence

The Chlamydomonas genome is GC-rich and the codon usage is highly biased toward a C in the third position. It is known for more than two decades that transgenes should adopt the codon usage of Chlamydomonas (Fuhrmann et al., 1999), which represents a more crucial factor for enhanced transgene expression than simply a high GC content (Barahimipour et al., 2015; Weiner et al., 2018). The use of preferred codons results in high transcript levels and translational efficiency (Barahimipour et al., 2015; Weiner et al., 2018).

## Transgene Design: The Addition of Introns

As for the codon usage, it is known for decades that inserting introns into transgenes contributes to enhanced transgene expression (Lumbreras et al., 1998; Fuhrmann et al., 1999). The intron most frequently used is the first intron of the *RBCS2* gene (*RBCS2i1*) which was postulated to contain an enhancer of expression (Lumbreras et al., 1998; Fuhrmann et al., 1999), although this was not confirmed later (Baier et al., 2020). In addition to the possible presence of transcriptional enhancers, introns are likely to facilitate high levels of gene expression, in a process called Intron-Mediated Enhancement (IME; Schroda, 2019; Baier et al., 2020). This IME was investigated by Baier et al. (2020), who showed that other introns than *RBCS2i1*, such as the *LHCBM1i2* intron, are responsible for IME, either because the intron would stimulate transcription or because the spliceosome would facilitate the interaction with the RNA polymerase for efficient transcriptional elongation. (Schroda, 2019; Baier et al., 2020). The strategy to obtain high transcription levels is to interrupt transgenes with introns at regular intervals. An online tool is available to design transgenes with an appropriate number of introns (Jaeger et al., 2019).

## The Chlamydomonas Modular Cloning kit (MoClo)

**Figure 1:**
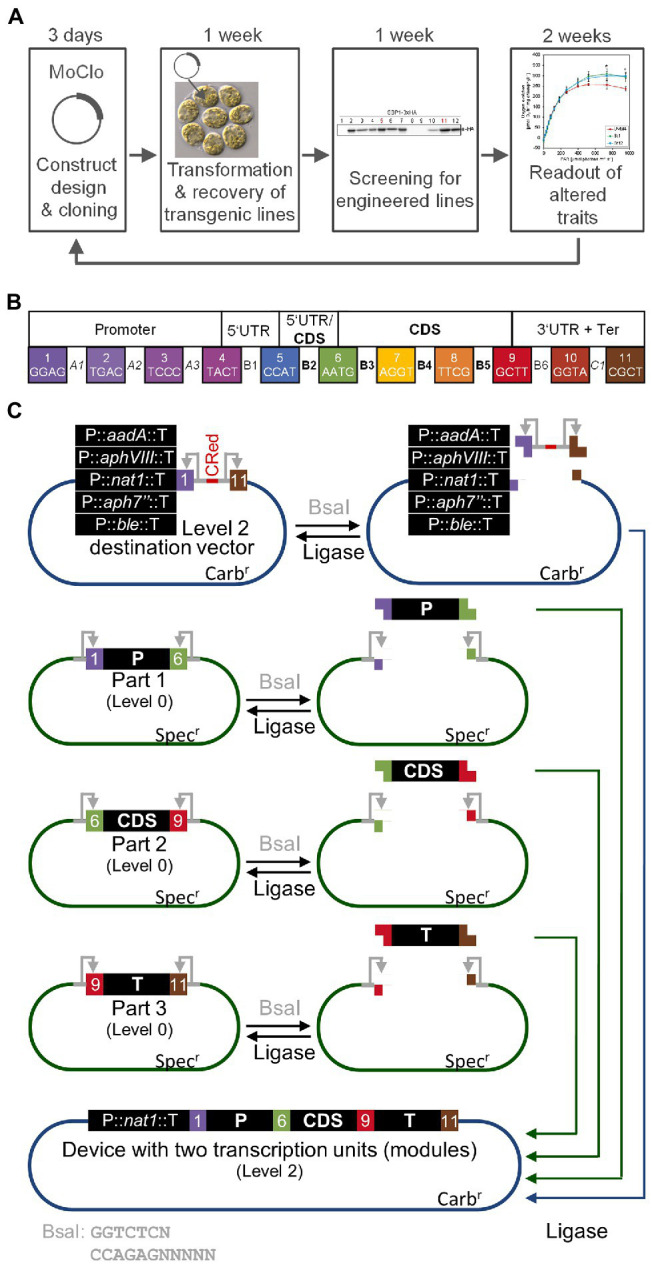
The MoClo strategy in Chlamydomonas. **(A)** The iterative cycle of transgene design, building, testing, and learning (DBTL) can be achieved in 4–5 weeks with Chlamydomonas. **(B)** Plant MoClo syntax with the 11 fusion sites (4-nt overhangs) flanking the positions (A1–C1) for the individual parts of a transcription unit indicated by the color code (Patron et al., 2015; Crozet et al., 2018). **(C)** Directional assembly of a transcription unit (or module) into dedicated Chlamydomonas level 2 destination vectors. Standardized genetic parts are released from level 0 vectors by digestion with BsaI (gray). Exemplarily, three parts representing promoter (P, position A1-B2), coding sequence (CDS, position B3-B5), and terminator (T, position B6-C1) are shown. Recognition sites for BsaI remain on the vector (gray arrows). In the same reaction, one of five dedicated level 2 destination vectors is digested. Here, the BsaI recognition sites are released with the insert containing the bacterial CRed operon for canthaxanthin biosynthesis allowing red/white color selection. Since T4-DNA ligase is present in the same reaction, genetic parts can be religated into their level 0 source vector and the insert can be religated into the level 2 destination vector. However, both can be cut out again. FIGURE 1Level 2 destination vector and released genetic parts contain compatible overhangs that allow a directional assembly of the parts into the level 2 destination vector. Once this has occurred, the parts cannot be cut out again, since the BsaI recognition sites are lost, explaining the high efficiency of the assembly reaction with multiple parts. The digestion/ligation reaction proceeds for several hours and products are then transformed into *E. coli* followed by plating on carbenicillin to select for white colonies harboring assembled level 2 vectors. The five dedicated level 2 destination vectors already contain cassettes conferring Chlamydomonas cells resistance to commonly used antibiotics [*aadA*—spectinomycin (Meslet-Cladière and Vallon, 2011), *aphVIII*—paromomycin (Sizova et al., 2001), *nat1*—nourseothricin (Yang et al., 2019), *aph7”*—hygromycin (Berthold et al., 2002), *ble*—phleomycin (Stevens et al., 1996)]. Therefore, the transcription unit assembled in a single reaction can directly be transformed into Chlamydomonas.

As outlined above, nuclear transformation of Chlamydomonas is easy and transformant colonies can be recovered in less than a week. Thanks to the short doubling time of Chlamydomonas of 6–8 h, the cultivation of transformed lines and screening for expressors takes only another week. Likewise, the production of sufficient biomass for the analysis of traits conferred by the transgene can be realized in another 2 weeks, overall allowing for the testing of a transgene construct in as little as 4 weeks (Figure 1A). The bottleneck was the cloning of the construct by classical cloning techniques. These typically allow the combining of only two genetic parts at a time with low efficiency. Moreover, since classical cloning is based on a variety of type II restriction enzymes, genetic parts are not standardized and thus not interchangeable. These problems were recently solved for Chlamydomonas by introducing a synthetic biology approach based on Modular Cloning (MoClo; Crozet et al., 2018). MoClo uses standardized genetic parts and standardized part assembly routes (Weber et al., 2011; Patron et al., 2015). Efficient assembly of several predefined genetic parts in a single reaction is achieved by Golden Gate cloning using the type IIS restriction enzymes BsaI and BpiI as well as T4 DNA ligase. Standardization of the genetic parts means that they must lack BpiI and BsaI recognition sites and are cloned into specific, so-called level 0 destination vectors. BsaI digestion of these vectors releases the genetic parts with characteristic 4-nt overhangs (Figure 1B). These represent defined fusion sites flanking the functional parts of a transcription unit (promoter, 5’-UTR, signal peptide, CDS, tag, 3’-UTR, terminator). These parts are then directionally assembled into a complete transcription unit within a level 1 destination vector present in the restriction/ligation reaction. In a second step, several transcription units in level 1 vectors can be released by cleavage with BpiI and assembled in a single step into a level 2 destination vector present in the same reaction. This second assembly step allows the construction of multigene clusters that, for example, encode the enzymes responsible for a complete metabolic pathway. The original Chlamydomonas MoClo toolkit contains 119 standardized parts including promoters, targeting signals, reporters, affinity tags, terminators, and much more (Crozet et al., 2018). Many new parts were added since then (de Carpentier et al., 2020; Mehrshahi et al., 2020; Dementyeva et al., 2021; Einhaus et al., 2021; Geisler et al., 2021; Kiefer et al., 2021; Niemeyer et al., 2021) and it can be anticipated that the kit will continuously grow. Very often, single target genes are tested in Chlamydomonas, for example if mutants are to be complemented (Theis et al., 2019; Spaniol et al., 2022). This is particularly relevant considering that an insertional mutant library generated by the Chlamydomonas Library project (CLiP) and containing ~60,000 strains, is available for the community (Li et al., 2016). For complementation, the target gene and a resistance cassette must be assembled into a level 2 vector. To spare the normally required two assembly steps, level 2 recipient vectors were constructed that already contain five commonly used resistance cassettes and allow the one-step assembly of the target gene from level 0 genetic parts (Figure 1C; Niemeyer and Schroda, 2022).

## The CRISPR/Cas9 Technology for Genome Editing

**Figure 2:**
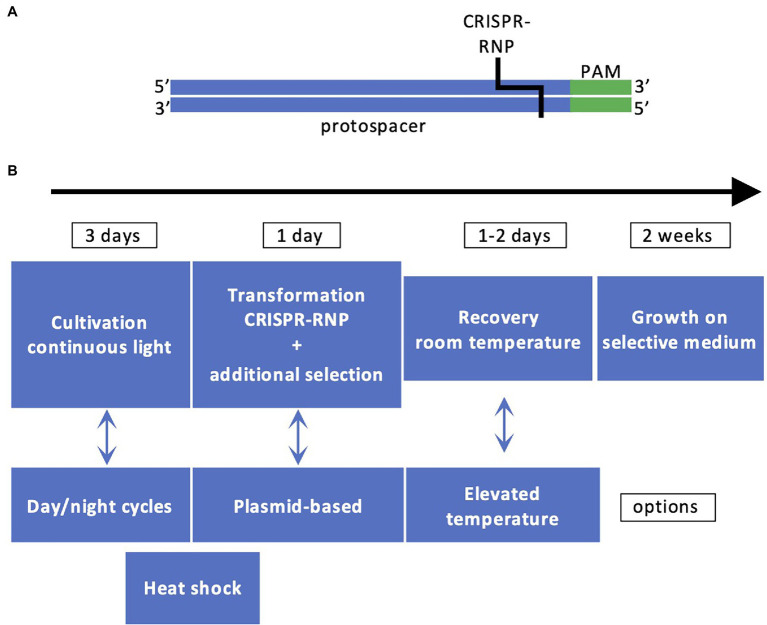
Principles of CRISPR/Cas9 technology in Chlamydomonas. **(A)** The DNA recognition sequence comprising the protospacer (in blue) and PAM (in green) is recognized by CRISPR-RNP. **(B)** Workflow for generating CRISPR/Cas9 mutants. The most employed workflow comprises cultivation of cells under light, cotransformation by electroporation with CRISPR-RNP and a resistance cassette with homologous arms or ssODN for targeted insertion, cell recovery, selection based on antibiotic resistance and/or phenotype, and molecular characterization by PCR.

Successful demonstrations of the CRISPR/Cas9 technology in Chlamydomonas were published in 2016 (Baek et al., 2016; Shin et al., 2016). The CRISPR/Cas9 technology is a simple method to produce sequence-specific genomic breaks. The Cas9 protein is an RNA-guided nuclease. The RNA sequence associated with Cas9 comprises two parts, one called tracrRNA that interacts with Cas9 and a single guide RNA (sgRNA) specific to the DNA target. The protospacer adjacent motif (PAM) is a DNA sequence (2–6 bp) that follows the DNA region targeted by the sgRNA (Figure 2). When Cas9 recognizes PAM, it checks the upstream region. If this region is marked by the sgRNA, Cas9 introduces a double-strand break 3–4 nt upstream of PAM, in the sequence corresponding to the sgRNA. The PAM is specific to the bacterial species (Ghribi et al., 2020). The sgRNA is designed using web tools which inform about the number of possible off-targets. The traditional way to deliver the sgRNA and Cas9 is plasmid-mediated. This is not the case in Chlamydomonas where the sgRNA is synthesized *in vitro*, assembled with the tracrRNA and delivered together with Cas9 by electroporation in a CRISPR-Ribonucleic Protein complex (CRISPR-RNP). This way of delivering Cas9 in a CRISPR-RNP complex to the Chlamydomonas cells was proven to be more efficient than expressing Cas9 by transgene expression which is proposed to induce cell toxicity (Jiang et al., 2014), although this method has also been shown to be successful later on Greiner et al. (2017) and Guzmán-Zapata et al. (2019). Another delivery system has also been recently proposed: it is still relying on a CRISPR-RNP complex but in addition, a cell-penetrating peptide is added to facilitate the entrance into the cells, which avoids the use of electroporation (Kang et al., 2020). The double-strand breaks made by Cas9 are repaired *via* the non-homologous end joining (NHEJ) repair pathway, which is error prone. The methodology for Chlamydomonas is described in Figure 2, due to the fast growth of Chlamydomonas, the success of the experiments can be evaluated in 3 weeks.

Some of the targeted genes, such as CpSRP43 and CpFTSY, are involved in the assembly of the antenna proteins and their impairment is easy to detect since it leads to loss of pigmentation of the algal cells (Baek et al., 2016; Shin et al., 2016). However, the mutation rate is low (~0.5% of the pale green colonies) and a co-transformation strategy was applied with vectors providing resistance to hygromycin B to increase the number of transformants (Shin et al., 2016). In that case, it was observed that the cassette could be integrated at the Cas9 cutting site. This led to an improvement of the method by the addition of flanking sequences homologous to the target gene (homologous arms) to both ends of the resistance cassette, which contributes to increase the number of positive transformants where the cassette is integrated at the site of the double-strand break induced by Cas9 (Angstenberger et al., 2020; Picariello et al., 2020). Editing frequency was also affected by other factors (Figure 2). A heat shock pretreatment of the cells before or after the electroporation was shown to increase the rate of mutations (Greiner et al., 2017; Dhokane et al., 2020; Akella et al., 2021), as well as cell synchronization (Angstenberger et al., 2020). A co-targeting strategy was recently designed to allow precise editing. In this case, two CRISPR-RNPs were co-electroporated, both with the classical sgRNA for each target gene and with single-stranded oligodeoxyribonucleotides (ssODNs) to allow precise edits (Akella et al., 2021). One of the target genes was *PPX1* encoding protoporphyrinogen IX oxidase. The ssODN of *PPX1* contained a point mutation conferring resistance to herbicides in Chlamydomonas. The second gene was the *FTSY* gene mentioned above and the ssODN contained a STOP codon. A few pale green colonies with the desired STOP codon were recovered among the herbicide resistant colonies, which opens the way to create strains with precise mutations in several target genes (Akella et al., 2021). The increasing number of reports based on CRISPR/Cas9 in Chlamydomonas is a proof that this technique is effective (Ghribi et al., 2020) and could represent a method of choice for optimizing fluxes through desired biosynthetic pathways.

## Examples and Outlook

In the following we give four examples for the use of Chlamydomonas as a chassis for the production of biotechnologically interesting compounds based on the recent improvements outlined above. In all examples, except the last one, strains with defects in the *SRTA* gene were used, as well as codon-adapted genes, harboring *RBCS2* introns, driven by the *HSP70A-RBCS2* promoter. The first two examples also employed the MoClo strategy. In the first example, Freudenberg et al., 2021 produced a bacterial lysine decarboxylase fused C-terminally with YFP or RFP, a 111-kDa protein, for the production of cadaverine (Freudenberg et al., 2021). Cadaverine serves as a precursor for the synthesis of (bio)polyamides such as polyamide plastics. Using a modified growth medium allowing for cell densities of up to 2 × 10^8^ cells/ml, the authors obtained significant cadaverine yields with the cytosolically expressed enzyme. However, yields were ~10-fold lower than with bacterial production systems, with limitations imposed by the toxicity of high cadaverine concentrations (Freudenberg et al., 2021). In the second example, Kiefer et al. (2021) produced and secreted the full-length ~137-kDa SARS-CoV-2 spike protein lacking its C-terminal transmembrane domain. As judged from its solubility and ability to interact with the human ACE2 receptor, the Chlamydomonas-produced protein was functional (Kiefer et al., 2021). This is surprising, given the presence of 22 N-glycans on the protein and the different composition of glycans on secreted proteins in Chlamydomonas and humans (Mathieu-Rivet et al., 2020). However, problems with low yields and the purification of the protein from the medium still need to be solved (Kiefer et al., 2021). In the third example, Perozeni et al. (2020) overexpressed a modified form of the endogenous β-carotene ketolase in the chloroplast, resulting in a conversion of up to 50% of native carotenoids into astaxanthin and more than 70% into other ketocarotenoids (Perozeni et al., 2020). Although astaxanthin levels were clearly below those obtained with the natural astaxanthin producer *Haematococcus pluvialis*, the accessibility of the pigments is much better in Chlamydomonas (Perozeni et al., 2020). In the fourth example, Song et al. (2020) employed the CRISPR/Cas9 technology to generate a double mutant knockout in the zeaxanthin epoxidase and the lycopene epsilon cyclase genes. This led to drive the lycopene flux to zeaxanthin, by eliminating the branch to lutein and would facilitate the production of highly pure zeaxanthin (Song et al., 2020).

These examples demonstrate that recent developments including strain improvement, insights into gene design requirements, ease of construct building *via* MoClo, and the establishment of CRISPR/Cas9 for genome editing have boosted the potential of Chlamydomonas for genetic engineering. These will pave the way for Chlamydomonas as a platform for sustainable green biotechnology.

## Author Contributions

All authors listed have made a substantial, direct, and intellectual contribution to the work and approved it for publication.

## Funding

Research in the laboratory of MS is supported by the Deutsche Forschungsgemeinschaft (SPP 1927, Schr 617/11-1 and TRR 175, project C02) and in the laboratory of CR by Fonds National de la Recherche Scientifique (FNRS; CDR J.0175.20); Fonds Wetenschappelijk Onderzoek—Vlaanderen (FWO) and FNRS under the Excellence of Science (EOS) Project No. 30829584; and Action de Recherche Concertée from the University of Liege (DARKMET ARC grant 17/21-08).

## Conflict of Interest

The authors declare that the research was conducted in the absence of any commercial or financial relationships that could be construed as a potential conflict of interest.

## Publisher’s Note

All claims expressed in this article are solely those of the authors and do not necessarily represent those of their affiliated organizations, or those of the publisher, the editors and the reviewers. Any product that may be evaluated in this article, or claim that may be made by its manufacturer, is not guaranteed or endorsed by the publisher.
